# Crystal structure of a tripartite complex between C3dg, C-terminal domains of factor H and OspE of *Borrelia burgdorferi*

**DOI:** 10.1371/journal.pone.0188127

**Published:** 2017-11-30

**Authors:** Robert Kolodziejczyk, Kornelia M. Mikula, Tommi Kotila, Vincent L. G. Postis, T. Sakari Jokiranta, Adrian Goldman, Taru Meri

**Affiliations:** 1 Division of Biochemistry, Department of Biosciences, University of Helsinki, Helsinki, Finland; 2 Institute of Biotechnology, University of Helsinki, Helsinki, Finland; 3 Biomedicine Research Group, Faculty of Health and Social Sciences, Leeds Beckett University, Leeds, United Kingdom; 4 Research Programs Unit, Immunobiology, University of Helsinki, Helsinki, Finland; 5 Astbury Centre for Structural Molecular Biology, School of Biomedical Sciences, Faculty of Biological Sciences, University of Leeds, Leeds, United Kingdom; 6 Division of General Microbiology, Department of Biosciences, University of Helsinki, Helsinki, Finland; University of North Dakota School of Medicine and Health Sciences, UNITED STATES

## Abstract

Complement is an important part of innate immunity. The alternative pathway of complement is activated when the main opsonin, C3b coats non-protected surfaces leading to opsonisation, phagocytosis and cell lysis. The alternative pathway is tightly controlled to prevent autoactivation towards host cells. The main regulator of the alternative pathway is factor H (FH), a soluble glycoprotein that terminates complement activation in multiple ways. FH recognizes host cell surfaces *via* domains 19–20 (FH19-20). All microbes including *Borrelia burgdorferi*, the causative agent of Lyme borreliosis, must evade complement activation to allow the infectious agent to survive in its host. One major mechanism that *Borrelia* uses is to recruit FH from host. Several outer surface proteins (Osp) have been described to bind FH *via* the C-terminus, and OspE is one of them. Here we report the structure of the tripartite complex formed by OspE, FH19-20 and C3dg at 3.18 Å, showing that OspE and C3dg can bind simultaneously to FH19-20. This verifies that FH19-20 interacts *via* the “common microbial binding site” on domain 20 with OspE and simultaneously and independently *via* domain 19 with C3dg. The spatial organization of the tripartite complex explains how OspE on the bacterial surface binds FH19-20, leaving FH fully available to protect the bacteria against complement. Additionally, formation of tripartite complex between FH, microbial protein and C3dg might enable enhanced protection, particularly on those regions on the bacteria where previous complement activation led to deposition of C3d. This might be especially important for slow-growing bacteria that cause chronic disease like *Borrelia burgdorferi*.

## Introduction

Complement (C) is an important part of the human innate immunity system. Over 30 proteins of the C system interact with each other leading to a cascade-like activation that terminates either in phagocytosis of the target or the formation of the lytic membrane attack complexes [1]. The major targets for C activation are microbes, but C also participates in immune complex removal and clearance of apoptotic cells.

Target recognition, which precedes C activation, occurs *via* one of three pathways: the lectin, classical and alternative pathways. Binding of mannan-binding lectin to mannose or other carbohydrates on the target surface leads to lectin pathway activation. The classical pathway recognizes surfaces when C1q binds to Fc domains of multiple antibodies attached to targets[2]. The alternative pathway (AP) is activated by C3b, which randomly and covalently attaches to primary amine and hydroxyl groups on a target surface *via* a thioester group located in the thioester-containing domain (TED). C3b thus can cross-link to any surface lacking C regulators. To protect host cells, and to prevent depletion of C protein, several proteins on the cell surface and in the fluid phase inhibit C activation. Regulation of the AP activation is especially important as the target recognition is mediated *via* interplay between C3b, C regulators and properties of target surfaces.

The main AP regulator is factor H (FH). It is a 155 kDa glycoprotein found in plasma at 200 to 300 μg/ml concentration [3]. FH consists of 20 globular homologous domains called complement control protein modules (short consensus repeats. SCRs or sushi-domains). Domains 1 to 4 bind to the main complement protein C3b and mediate the regulatory functions [4–6]. The FH-C3b complex recruits the protease factor I which cleaves C3b leading to its inactivation (cofactor-activity). FH can also regulate the formation of C3b-activating convertases of the AP (C3bBb) by competing with factor B in binding to C3b [7], and FH can additionally accelerate decay of the preformed convertases [8]. Cleavage of C3b by factor I leads to formation of smaller fragments iC3b and C3dg. The TED-domain is located in the C3dg domain, which means that even when C3b is cleaved, the C3dg fragment stays attached to the surface [9]. Surface bound cleavage fragments of activated C3 (especially the C3dg fragment) act as ligands for C receptor 2 (CR2), which is found on B-cells. If CR2 recognizes target-bound cleavage fragments simultaneously with B-cell co-receptor complex binding its antigen, subsequent B-cell activation is markedly enhanced [10]. CR2 is composed of SCR-domains like FH, and SCRs 1 and 2 mediate binding to C3d [11].

The FH protein family also includes FHL-1, an alternatively spliced variant of the *CFH*–gene and five FH-related proteins (FHRs), which are encoded by separate *CFHR1-5* genes. FHRs are different in size and consist of four, five or nine similar SCR-domains as FH [12]. Some of the domains are highly homologous with important ligand binding sites in FH, *eg*. domains 19–20 and 6–7 [13]. Even though all FHRs lack the functional domains 1–4, FHRs have been suggested to act as regulators of AP (for review, see [12], [14]). A recent structural study suggested that, by dimerization, three out of five FHRs could compete with FH in binding to different ligands and thus their major function would be to deregulate FH [14, 15]. By either competing with FH or showing direct regulatory functions themselves FHRs could modulate AP activation depending on local environment, *e*.*g*. composition of surfaces, concentration of proteins *et cetera*.

FH has been long known to have a critical role in C regulation, as it has been shown that single point mutations in FH cause severe diseases. For example, in atypical haemolytic uremic syndrome (aHUS), mutations in FH lead to cell damage, especially in the kidneys [16]. Furthermore, polymorphism of one amino acid in domain 7 is an important risk factor in development of age related macular degeneration, the most common cause of blindness in the elderly in industrialized countries [17].

The C-terminal domains 19–20 of FH (FH19-20) are important in several physiological interactions. These domains contain a glycosaminoglycan binding site [18] that mediates binding to endothelial cells [19], gangliosides [20], and malondialdehyde moieties on proteins [21]. We previously solved the structure of the C-terminus alone [22] and in complex with C3d [23]. Interestingly, the structure of the complex showed that C3d has two different binding sites on the C-terminus of FH: one in domain 19 and the other in domain 20. This dual binding property facilitates target discrimination of FH: while FH is bound to glycosaminoglycan containing surfaces *via* domain 20 it can simultaneously bind to the C3d part of the main C opsonin, C3b, *via* domain 19 ([23].

Bacteria, yeasts and even parasites utilize FH for protection against C [24]. Typically, microbes bind FH to their surfaces to prevent C activation. Two major interaction sites on the FH molecule have been identified, one in domain FH7 and the other in the C-terminus [25]. Various microbes, including Gram-negative and Gram-positive bacteria and yeast *Candida albicans* use the C-terminal site. By using a series of FH19-20 mutants in binding assays, the binding site of most of these microbes has been further localized to the side of domain 20, an area we called the “common microbial binding site” [25]. We have also shown that when FH is bound to a microbial protein *via* this site, its regulatory activity against C3b is enhanced. For instance, when domain 20 is occupied by OspE, activated C3b is cleaved into inactive iC3b faster [25].

Lyme disease or borreliosis is a zoonosis caused by different genospecies of spirochetal bacteria from the genus *Borrelia*. The infection is initiated through a bite of an infected *Ixodes* tick. The disease affects most commonly the skin and is usually limited to that. However, joints, nervous system and other locations can also be infected. *Borrelia* can persist in the host for long periods, partly due to the immune privileged sites they occupy, such as joints or the central nervous system, but also because they escape immune surveillance very efficiently. Without protection *Borrelia* are sensitive to human C [26]: C evasion is thus essential for these bacteria. *Borrelia* are able to recruit host FH [27] and another host C regulator, C4b binding protein [28].

Binding FH appears to be very important for *Borrelia*, as several FH-binding proteins or protein families have been identified. CspA (BBA68/CRASP-1) and CspZ (CRASP-2) bind FH and FHL-1 *via* FH7 and FH *via* FH19-20 [29]. OspE belongs to the family of borrelial proteins called Erps (OspE/F-related proteins). We recently solved the structure of OspE from the strain *B*. *burgdorferi* sensu stricto (strain N40) alone and in complex with FH19-20 [30]. The binding site we identified on FH20 was verified using a panel of 14 mutant proteins in binding assays [25]. There are several highly homologous proteins in the family and crystal structures of ErpP and ErpC have been recently solved [31, 32]. The structures are similar, except that there is a stretch of eight residues located between β-strands 3 and 4 in ErpC [32].

This article describes the crystallization and structure of the tripartite complex structure of OspE:FH19-20:C3dg at 3.18 Å to an R_work_/R_free_ of 23.3/26.06%. The structure revealed that FH19-20 can interact simultaneously with bacterial protein and the main protein of the C system, C3b. Binding to C3b is mediated *via* C3dg, as we previously suggested [23]. Our structural data suggest that the C-terminus of FH has a dual function: it can bind to surfaces and C3b simultaneously and facilitate down-regulation of C.

## Materials and methods

### Cloning, expression and purification of proteins

OspE (residues 21–171) from the strain *Borrelia burgdorferi* N40 was cloned, expressed and purified as previously described [30]. Wild-type FH19-20 was cloned, expressed in *Pichia pastoris* and purified using heparin affinity column as described [22]. C3dg (residues 955 to 1303) was recloned from the pET-15b vector [33] to pET-22b vector by restriction-free cloning using a forward primer C3dg-F and reverse primer C3dg-R. The forward primer introduced a hexa-histidine purification tag and a Tobacco Etch Virus (TEV) cutting site at the N-terminus. Using site directed mutagenesis, the A1153E and C1010A mutations were introduced to the gene with primer pairs: QC_A1153E-F and QC_A1153E-R, and QC_C1010A-F and QC_C1010A-R, in two consecutive reactions, to reverse the A1153E mutation in the original gene and to prevent thioester formation by introducing the C1010A mutation. We also recloned the His_6_-tag from the N-terminus to C-terminus in two steps using site directed mutagenesis and the following primer pairs: C3dg-2F and C3dg-2R (to remove His_6_-TEV from the N-terminus), C3dg-3F and C3dg-3R (to introduce TEV-His_6_ at the C-terminus). All primers used are listed in S1 Table.

### C3dg purification

The C3dg construct was expressed in *E*. *coli* BL21(DE3) grown in LB medium. Cells were grown at 37° C to an OD_600_ of 1.0–1.2, the temperature was decreased to 25° C and protein expression induced by adding 0.1 mM isopropyl β-D-1-thiogalactopyranoside. After 2 hours, cells were harvested by centrifugation at 6000 *g* at 10° C for 10 minutes. The cell pellet was resuspended in 50 mM sodium phosphate (pH 8.0), 500 mM NaCl, 10% (v/v) glycerol, and 20 mM imidazole, and frozen at -80° C. After being thawed, the cells were sonicated 3 x 3 min (60% of total power) using a tip sonicator on an ice bath. After centrifugation (4° C, 30 min, 30,000 *g*), the supernatant was loaded onto a 4 ml Ni-NTA column, and the bound protein was eluted with 300 mM imidazole. Fractions containing C3dg were pooled and buffer exchanged to 50 mM sodium phosphate (pH 8.0) with 150 mM NaCl, 10% (v/v) glycerol. For His-tag removal, TEV protease added at a ratio of 100:1 (w/w). After incubation at room temperature overnight, the cleaved tag and the protease were removed by negative purification on a 4 ml Ni-NTA column. A polishing purification step was performed using a HiLoad 26/600 Superdex 200 column equilibrated with 20 mM Tris pH 7.5, 150mM NaCl. Finally, the protein was concentrated using an Amicon concentrator with a 10 kDa cutoff to 10 mg/ml. The purity was tested with SDS-PAGE and was above 95%.

### Crystallization, data collection and structure analysis

The OspE:FH19-20:C3dg complex was crystallised at 20° C at a 1:1:1 molar ratio by mixing defined amounts of the three purified proteins together prior to crystallisation. The reference concentration was 10 μl of 10.7 mg/ml of C3dg and the crystallization trials used sitting drop vapour diffusion in 200 nl drops (100 nl of protein solution and 100 nl of well solution). Needle- and plate-like crystals appeared in the Helsinki Random Screen 2 (HR2). Hit conditions from HR2 (D9: 25% PEG 3350, 0.1 M Tris pH 8.5; G9: 25% PEG 3350, 0.1 M Tris pH 8.5, 0.2 M ammonium acetate; G12: 25% PEG 3350, 0.1 M Hepes pH 7.5, 0.2 M MgCl_2_) were optimised manually by preparing hanging drops with 2 μl of protein and 2μl reservoir. Harvestable 3D crystals appeared within two weeks from the following conditions: 20% PEG3350, 0.1 M Hepes pH 7.5, 0.2 M MgCl_2_ or 24% PEG3350, 0.1 M Hepes pH 7.5, 0.2 M MgCl_2_ or 16% PEG3350, Tris pH 8.5, 0.2 M ammonium acetate. Samples were cryo-protected by soaking in mother liquid containing 25% PEG400. The diffraction data were collected at Diamond Light Source synchrotron, on the I24 beamline at 100 K on a Pilatus3 6M detector. 1800 images were collected at an oscillation angle of 0.1°. Data were merged and scaled using X-ray Detector Software (XDS) [34]. Molecular replacement was done using Phaser [35] from the Phenix package [36] and published structures of OspE (PDB 4J38), FH19-20 (PDB 4J38) and C3dg (PDB 2XQW) as search models. Analysis of the Matthews coefficient based on unit cell parameters and the protein molecular weight of the tripartite complex pointed to two (3.04 Å^3^ Da^−1^) or three trimers (2.03 Å^3^ Da^−1^) in the asymmetric unit (ASU), with the highest probability (p (tot) = 0.62) pointing for the latter. C3d was used as a first search model and two or three molecules were searched in two separate runs. Results strongly suggested the existence of three C3dg molecules in ASU, and that solution was used in the next round to find the positions of three FH molecules. Then the C3d and FH solutions were used to search for OspE in the structure.

The structure was built and refined with phenix.refine and Coot [37]. Final model building and refinement to convergence was performed using BUSTER with grouped B-factors, Translation/Libration/Screw parametrization of a single group per chain first with all chains present (Rwork 0.2105, Rfree 0.2482). B-factors were calculated using CCP4 baverage program [38]. Structure figures were done using PyMOL (version 1.7, Schrödinger, LLC, New York). For rotation angles alignments were done in PyMol, using the Align routine with default settings. Two rotation matrices were output from PyMol, the FH19-alignment matrix (R_19_) and the FH20-alignment matrix (R_20_) and angles were calculated in Excel by using R_19→20_ = R_19_^-1^*R_20_, and calculating the rotation angle from the trace of the R_19→20_ matrix, a. Interfaces were analyzed using PISA [39].

## Results

### Crystallization, structure solution and refinement

**Fig 1:**
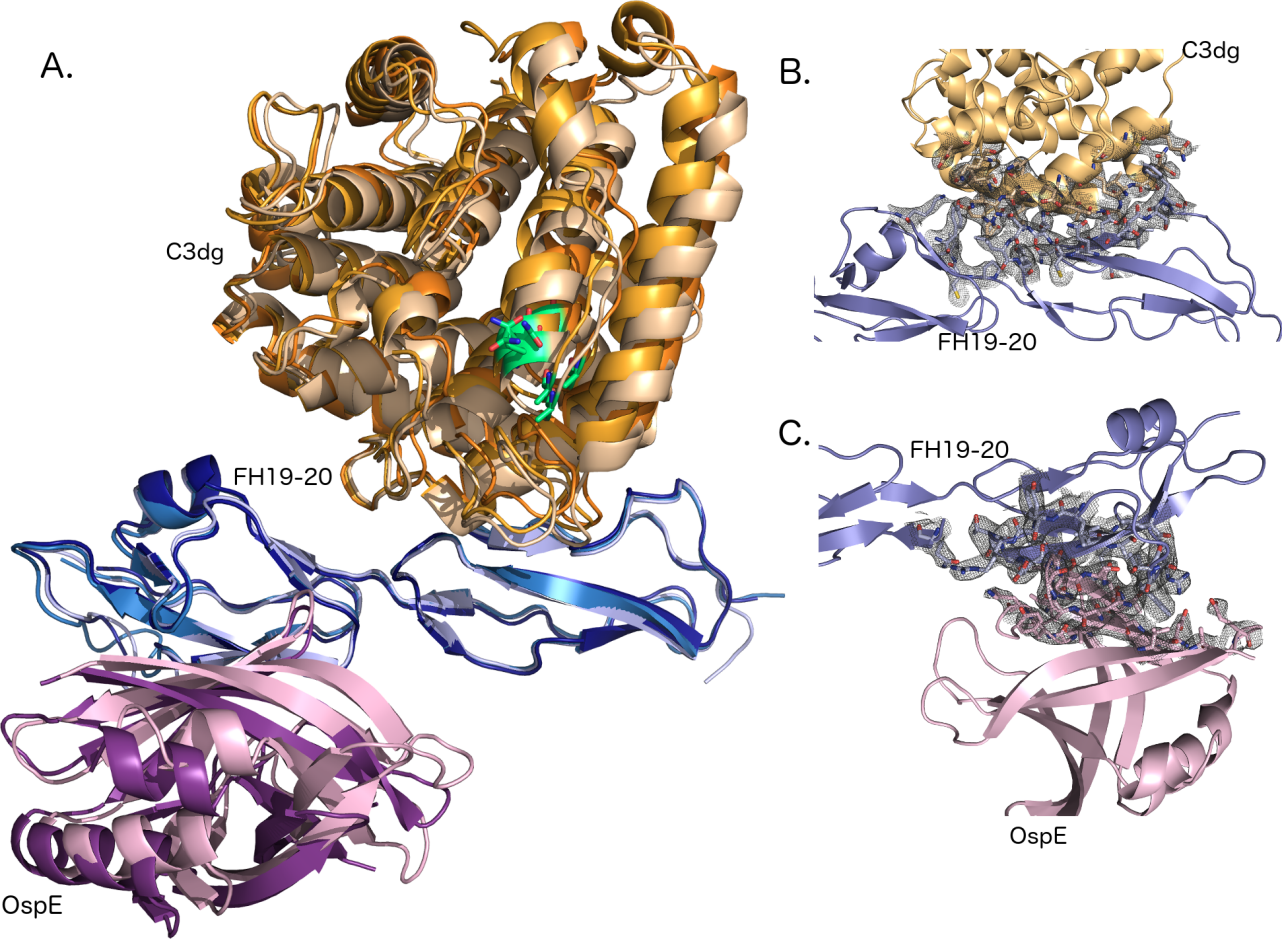
Overall architecture and interfaces of the complex. (A) C3dg on top (chain A: light orange, chain B: orange, chain C: dark orange), FH19-20 across (chain D: light blue, E: sky blue, F: deep blue) and OspE on the bottom (chain G: pink, I: violet purple). FH is sandwiched between C3dg and OspE and binds C3dg mainly *via* domain 19 and OspE on the opposing site *via* domain 20. Position of the thioester and amino acids involved is C3dg is marked using green. (B) A σA weighted electron density map of the binding interface between C3dg and FH19. A cartoon model of C3dg, chain A, is shown in orange on top and FH19-20, chain D, below in blue. Residues involved in binding are shown with electron density around them. (C) A σA weighted electron density map of the binding interface between and FH20 and OspE. FH, chain D is above and OspE, chain G is below. Residues involved in binding are shown as stick model in the electron density.

To determine the molecular mechanism of FH-mediated target discrimination on the bacterial surface, we solved the crystal structure of a tripartite complex formed by the C3dg part of complement C3, the C-terminus of human factor H (FH19-20) and outer surface protein OspE of *Borrelia burgdorferi* (strain N40). The complex crystallized in 4 μl sitting drops and single crystals were observed after two weeks (see Methods). The diffraction data were collected to 3.18 Å resolution and the structure was solved by molecular replacement using published structures of C3d:FH19-20 and FH19-20:OspE as search models. The structure was refined using BUSTER to R_work_/R_free_ values of 23.3% and 26.1%, respectively (Table 1). Electron density maps of all modeled chains (A-I) are shown in S1 Fig. The overall architecture of the complex and representative sections of the electron density maps from the binding interfaces between FH19-20 and C3dg and FH19-20 and OspE are shown in Fig 1.

**Table 1:** Data collection and refinement statistics.

Space group	P 2_1_2_1_2
Unit cell dimensions a, b, c (Å)	84.2, 124.9, 165.1
Molecules/asymmetric unit	3 heterotrimers*
Wavelength (Å)	0.969
Resolution limits	29.6–3.18
Temperature	100 K
Number of total reflections	186 606
Number of unique reflections	29 071
Completeness (%)	96.9 (82.3)**
Redundancy	6.4 (5.4) **
I/sigma(I)	12.4 (1.7)**
Resolution range	29.6–3.18
R_meas_	14.7 (98.3)
CC_1/2_	99.7 (67.0)
R_work_/R_free_ %	23.33 / 26.06
Number of atoms	11 754
Number of residues	1504
Number of water molecules	2
Root mean square deviation (RMSD) bonds (Å)	0.0091
Root mean square deviation (RMSD) angles (°)	1.10
Average B-factors (protein) (Å^2^)	91.13

*H-chain (OspE) from the trimer BE(H) was not modeled in the final structure because the electron density was too weak to trace

**in parentheses for the highest resolution shell.

### Identifying the structurally relevant unit

In our structure one asymmetric unit contains three closely packed heterotrimers with a water content of 48.8%. We first identified three C3dg-molecules and named them to chains A, B and C. The next protein identified was FH19-20, which we labeled D, E and F. The last protein in three trimers was OspE and that was labeled G, H and I. Thus, the first heterotrimer consists of chains ADG, the second BEH and third CFI. When building the structure into the electron density map, it was clear that some parts of the polypeptide chains did not have good density, even after many rounds of refinement and with the overall model having reached convergence. We therefore deleted small parts of chains E (1209–1211), F (1210–1212) and I (151–154) as there was no backbone density, and took the decision to delete chain H (OspE) totally. This was because, although there were small stretches of electron density indicating that it was present and adopted the same conformation as chains G and I, the overall density was so poor that we would have had at least 8 chain breaks, and there was almost no side-chain density at all. Leaving it out had only a minor effect on the R-factors (values with all the chains R_work_ 0.211, R_free_ 0.248 *versus* after deletion of the H-chain 0.2333 and 0.2606, respectively), and so we decided to omit it. From density it can be seen that chain H is clearly present, presumably at low occupancy and/or disordered (S1 Fig). For PDB-deposition the H-chain was replaced with a short alanine polypeptide chain allowing the assignment of H-chain sequence to the structure deposition. Thus, for PDB-users it is clear that H-chain most likely is present but not modeled and completes the third trimer.

### Crystal packing contacts

To identify the structurally relevant arrangement, the interface areas and numbers of hydrogen bonds between proteins were calculated using the PISA server from symmetry mates within 12Å (Table 2) [39]. Several potential contacts between C3dg, FH19-20, and OspE were found and assigned with corresponding polypeptide chains: A:D, D:G, B:E, C:F, F:I, A:E, B:F and B:C (Table 2). The first criterion for the interface to be physiologically relevant was that it had a buried surface area >400 Å^2^; only interfaces larger than this were analyzed in more detail.

**Table 2:** Macromolecular interfaces in the C3dg:FH19-20:OspE complex. Physiologically relevant interactions are shown with a grey background. A:D:G is trimer 1; B:E:(H) is trimer 2; C:F:I is trimer 3. n.a. = data not available.

Chain				
C3dg	FH19-20	OspE	Interface areas (Å^2^)	Number of Hydrogen bonds
A	D	G	A:D 829Å^2^, D:G 732Å^2^	16; 17
B	E	n.a.	B:E 825Å^2^	17: n.a.
C	F	I	C:F 830Å^2^, F:I 674Å^2^	14; 13
A	E		591Å^2^	10
B	F		571Å^2^	5
B	C		402Å^2^	4

The second criterion was the position of the thioester in the TED domain within C3d. Under physiological conditions, the thioester bond is exposed and enables interaction with the target surface. When C activates, the most likely order of events is attachment of C3b to a cell surface *via* the thioester in the C3d-domain, followed by recruitment of FH. Based on this, the A:E and B:F interfaces are non-physiological, as in their arrangement the thioester is blocked by FH. The B:C interface is between two C3dg-molecules, and likewise has no physiological relevance.

Consequently, three tripartite arrangements were selected for further analysis: A:D:G (trimer 1), B:E:(H) (trimer 2), C:F:I (trimer 3). All chosen interfaces are above 600 Å^2^ with more than 10 hydrogen bonds per interface (Table 2).

### Overall architecture of the heterotrimer

The first, the middle and the last chain in each tripartite complex correspond to the C3dg, FH19-20, and OspE molecules, respectively (Table 2). In all three C3dg chains, the N-terminal forty amino acids of C3dg were disordered.

We first superimposed of all three trimers upon each other, which revealed very minor differences in spatial organization (Fig 1). The root mean square deviations (RMSD) per Cα for the individual proteins were between 0.25 and 0.69 Å (Table 3). Two full trimers, A:D:G and C:F:I and proteins were compared to each other.

**Table 3:** Superimposition of trimers and proteins on each other. Cα’s of each proteins were compared to each other using align function in PyMol. Values shown are RMSD/Cα expressed in angstroms (Å). In parentheses used vs. total number of Cαs after five cycles is shown. Bold marks comparison of two full trimers.

	Trimer 2 (BE(H))	Trimer 3 (CFI)
Trimer 1 (ADG)	A:B 0.359 Å (269/291) D:E 0.360 Å(109/124)	**ADG:CFI 1.21 Å (426/535)** A:C 0.337 Å (256/291) D:F 0.667 Å (117/119) G:I 0.525 Å (123/125)
Trimer 2 (BE(H))		B:C 0.297 Å (262/293) E:F 0.603 Å (111/120)

The observed differences between the trimers result from dissimilarity in the relative orientation of the individual proteins, rather than the conformational changes thereof.

The main-chain temperature factors were calculated using B-AVERAGE in the CCP4-suite [34]. The mean values for C3dg chains A, B and C were 83 Å^2^, 77 Å^2^ and 82 Å^2^, respectively. For FH19-20, the values were slightly higher (for chains D, E and F 79 Å^2^, 81 Å^2^ and 96 Å^2^, respectively) and for the two chains of OspE 124 Å^2^ and 109 Å^2^.

When B-factors were plotted against the primary sequence, it was clear that the same molecules have a similar distribution of flexible and more rigid regions in the three different tripartite complexes (S2 Fig), even though each trimer experience a different environment in the crystal.

### Biological interactions

**Fig 2:**
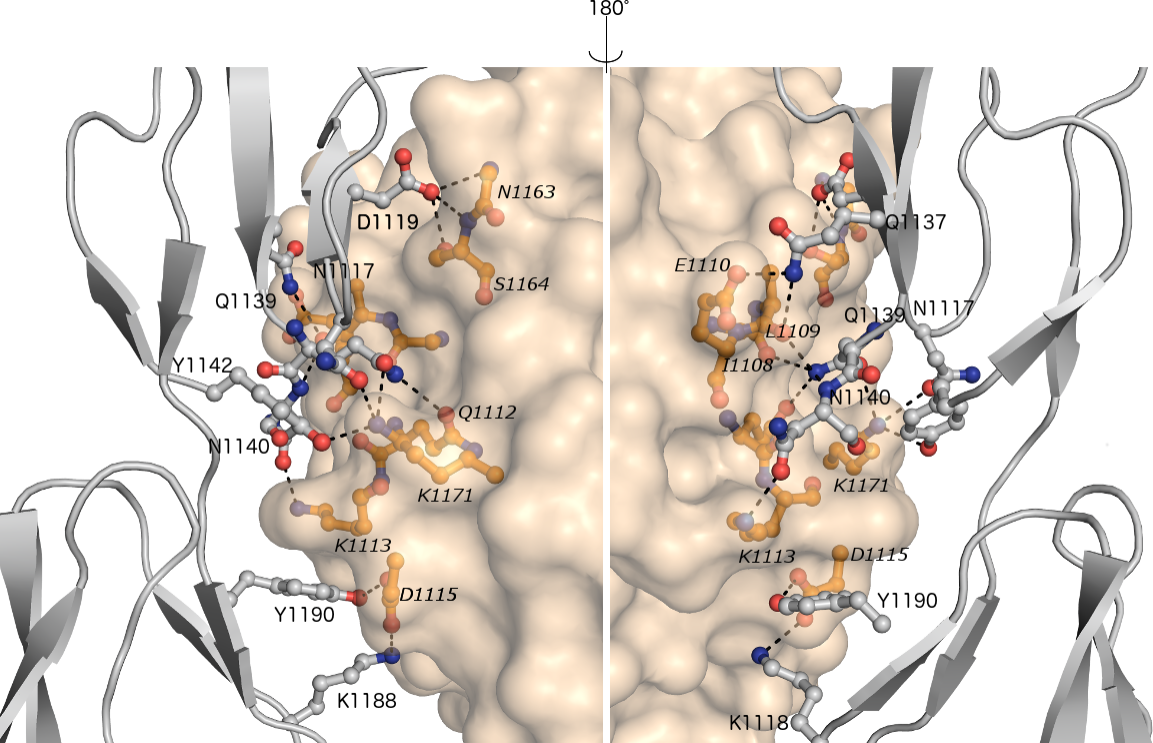
Binding interface between complement FH19-20 (chain F, grey cartoon) and C3dg (chain C, light yellow surface). Interface is shown from two directions about 180° apart with the interacting residues shown in ball-and-stick. The interface has 14 hydrogen bonds, marked using dashed lines between FH19 and C3dg. Residues in FH19 are italicised.

Previously published results suggested that FH19-20 binds OspE *via* domain 20 and C3dg *via* domain 19 [23], [25], [30]. Here we used the PISA server [39] to analyse interfaces and list hydrogen bonds in each heterotrimer. The contact surface area between FH and C3dg (Fig 2) is 830 Å^2^ and there are 14 hydrogen bonds in the C:F interface of the C:F:I trimer (Table 2 and S2 Table). FH binds to the side of the C3dg α/α barrel through helices α4 (residues 104–118) and α7 (residues 170–189). In C3dg residues I1108, L1109, Q1110, E1112, K1113, D1115 in helix 4, and N1163, S1164, K1171 in helix 7 form hydrogen bonds to FH19 (S2 Table). According to PISA analysis six of the hydrogen bonds can be found in our previously solved C3d:FH19-20 complex [23] (PDB 2XQW). Residues in FH that interact with C3dg all come from FH19 (N1117, D1119, I1120, S1122, Q1137, Q1139, N1140, Y1142, K1188, Y1190) and form a continuous patch to the site of the domain (Fig 2). The structure presented here strengthens our earlier structural and mutagenesis analysis [25], [30], suggesting that this is the biologically-relevant interface.

**Fig 3:**
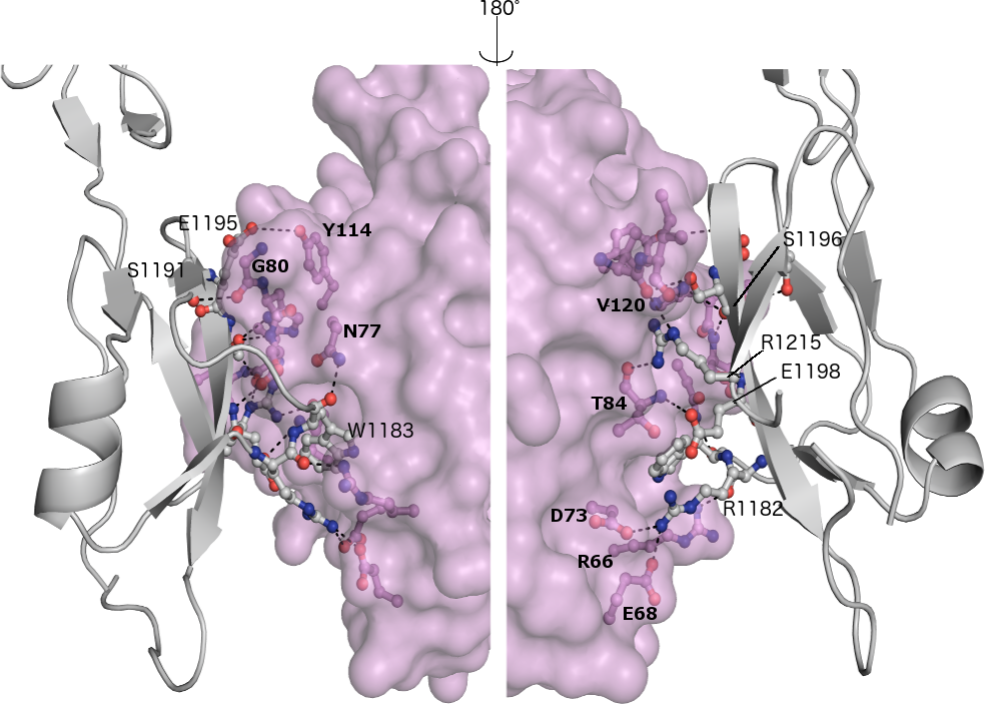
Binding interface between complement FH19-20 (chain F, grey cartoon) and OspE of *Borrelia burgdorferi* (chain I, pink surface). Interface is shown from two directions about 180° apart with the interacting residues shown in ball-and-stick. Binding is mediated *via* domain 20 of FH19-20. 13 hydrogen bonds in the interface are marked with dashed lines. Residues involved are annotated using a bold font for the amino acids from OspE and normal font for FH.

The contact area between FH and OspE in the C:F:I trimer is 674 Å^2^ (Fig 3) and contains 13 hydrogen bonds. On FH, the key amino acids R1182, E1195 and R1215 mediate binding to OspE loops β2, β3, and β4 and the interface between loops β5 and β6. There are some atomic level hydrogen bond differences in comparison with our previous heterodimer structure of FH19-20:OspE (S3 Table). The structure presented here as well as our earlier mutagenesis data [25] show that this is a biologically-relevant interface.

### Interesting differences

We next compared the C:F:I trimer to the previous structures of the same proteins. The individual proteins were very similar to the previously published structures when they were individually aligned. C3dg (chain C) was superimposed on the published structure (PDB 1C3D) [40] (0.252 Å/αC for 257 atoms after 5 cycles), OspE (chain I) on the published structure (PDB 4J38) (0.478 Å/αC for 257 atoms after 5 cycles), and FH19-20 (chain F) on PDB 2G71 (1.78 Å/αC for 257 atoms after 5 cycles). Removal of three residues of our FH chain F (1206–1208) due to poor density around them probably affected the RMSD value for FH. Comparisons to existing complexes gave similar results: FH19-20:C3d to PDB 2XQW [23] using FH19 binding site to C3d gave r.m.s.d value of 0.520 Å/αC for 351 atoms and comparison of FH19-20:OspE dimer from our complex to PDB 4J38 [30] gave RMSD 1.654 Å/αC for 234 atoms.

**Fig 4:**
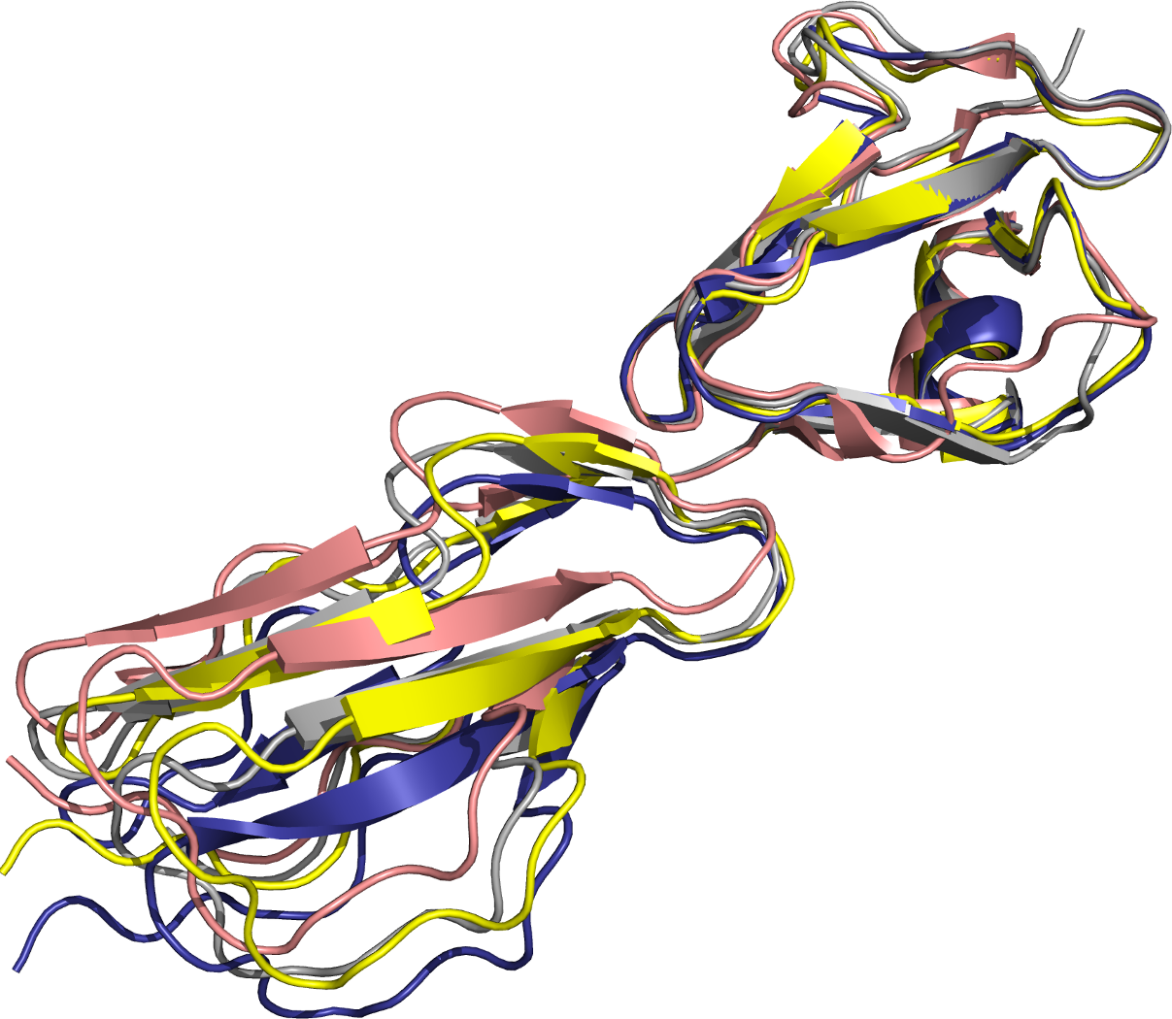
Superimposition of FH19-20 on previously published structures. FH19 (residues 1104–1164) from FH19-20 structure (PBD 2G71, pink [18] is aligned to chain F (deep blue), the published complex structures with OspE (4J38, yellow) [30] and with C3d (2XQW, grey) [23]. The orientation of FH20 residues 1201–1515 in the important target recognition loop is clearly different.

Despite the major similarities, there were some interesting differences when our tripartite structure was compared the earlier complex structures. We calculated rotation angles to be able to compare possible differences in FH20 orientation in non-bound and target-bound stages. We first aligned FH19s (residues 1105–1163) and calculated the angle which is required to rotate the FH20s (residues 1167–1228) to align optimally. The distance (Å) is the change in the centroid distance of FH20 upon the changed alignment. We compared all three FH chains (D, E and F) to each other with differences in rotation angles being less than 10° degrees. Next FH-chains were aligned with published structure of FH19-20 (PDB 2G7I) [18] and complex structures with OspE:FH19-20 (PDB 4J38)[30] and C3d:FH19-20 (2XQW) [19]. It can be seen that there was a pronounced shift in the relative orientation of FH20 with respect to FH19 when FH19-20 polypeptides were compared to the published structure of OspE:FH19-20. In addition, there was a smaller but different shift of FH20 with respect to FH19 when we compared polypeptide chains D, E and F to our earlier C3d:FH19-20 structure (PDB 2XQW) [23] (Fig 4 and Table 4).

**Table 4:** Rotation angles for FH domain 20 when domain 19 is superimposed. In the structure 4J38 FH is bound to OspE [30], in the 2XQW it is bound to C3d [23] and in the structure 2G7I [22] FH19-20 has no ligand. It can be seen that biggest differences in angles between chains E and F are to non-bound state of FH19-20 (2G7I).

	Chain D	Chain E	Chain F	PDB 4J38	PDB 2XQW
Chain E	2.24° 0.27 Å				
Chain F	8.64° 1.29 Å	6.69° 1.37 Å			
PDB 4J38 (OspE)	12.90° 3.37 Å	11.47° 3.04 Å	14.71° 3.79 Å		
PDB 2XQW(C3d)	5.28° 0.71 Å	5.01° 0.61 Å	11.94° 2.00 Å	8.77° 2.57 Å	
PDB 2G7I (no ligand)	18.64° 2.96 Å	21.01° 2.77 Å	25.6° 3.86 Å	17.03° 1.87 Å	13.27° 2.10 Å

## Discussion

Microbes need protection against C to be able to cause an infection. Several pathogens have evolved to use host C regulators to evade the attack of the innate immune system [23], but the molecular mechanisms have remained unclear until recently. The complement protein C3 is present in the bloodstream at approximately 1 mg/ml, allowing irreversible attack on non-protected surfaces within milliseconds of exposure [24]. Therefore, microbes need efficient C evasion mechanisms acting in tandem with C activation. For the successful recruitment of soluble regulators like FH, several criteria need to be fulfilled. First, FH needs to have access to the FH-binding molecule on the target surface. Our previous NMR structure of OspE showed that this molecule has a 50 Å long and very flexible N-terminal tail [30]. This allows a single OspE molecule to reach considerably from its site of attachment on the outer membrane, thus enabling it to “hook” FH from the plasma.

A second requisite for C regulation is that the simultaneous binding of FH, OspE, and C3dg to each other would not lead to steric hindrance with domains 1–4 of FH, which interact with the C3b and are needed for regulatory functions [5]. In our tripartite structure, the functional part of FH (FH1-4) could simultaneously bind into a groove on C3b [5, 6], This was checked by superimposing C3dg onto the equivalent part of the full structure of the C3b:FH1-4 complex (PDB entry 2WII, [5]), and no direct steric hindrance was detected. However, the closest approach between the C-terminal residue of FH1-4 (Q205) and the N-terminal residue of FH19 (S1126) is just 3.4 Å, meaning that there must be some conformational changes in the C-terminus of FH4 and the N-terminus of FH19, or a conformational change in C3b, to accommodate the intervening 901 residues of FH.

The C-terminus of FH (domains 19 and 20) has multiple roles in C regulation and interestingly it now seems that both SCR’s are used during target recognition. Even without structural data it was known, from other C regulators consisting of repetitive SCR-like domains, that single domains are not functional [41]. Furthermore, flexibility in the linker regions between domains has been suggested to facilitate binding to different ligands [42, 43]. Here, we measured and compared the orientation between domains 19 and 20 (Table 4 and Fig 3) to the structure of FH C-terminus alone [22], FH19-20 in complex with OspE [30] and C3d [23] and to our tripartite complex. It was seen the orientation of FH20 was clearly affected ligand binding. How different tilt, skew and twist angles between domains 19 and 20 are compared to other SCR-proteins [43] remains to be analyzed, but it seems there is flexibility which most likely facilitates binding to different ligands.

FH has a crucial role in C target recognition. A hotspot for this lies within domain 20. Using deletion mutants and several different biochemical approaches it has been previously shown that FH20 binds to sialic acids [44], glycosaminoclycans and heparin [6], mouse glomerular endothelial cells [19] and microbes [25]. Recent structural data have enhanced our understanding of binding interfaces for some interactions in more detail, like for sialic acids [20]. Binding of FH to different ligands seems to be mediated by almost identical residues. Consequently, microbes have evolved to utilize an overlapping, but not identical, site to the host to recruit FH. This is either *via* expression of unique FH-binding proteins like OspE, or by modification of surface properties by sugars (*e*.*g*. sialylation of surface moieties by *Neisseria*) [44]. Furthermore, our structure and previous structural data suggest [20] that a similar tripartite complex is formed on microbial surfaces as on surfaces of sialylated host cells. Finally, recent data suggest that successful tripartite complex formation between FH, sialic acids and C3b is a key factor in preventing cell damage from C attack [45].

Surface bound C3d is a very potent adjuvant and links innate and adaptive immunity: when a B-cell recognizes its antigen on a target surface *via* the B-cell receptor and C3-fragments *via* CR2 at the same time, subsequent B-cell activation is up to thousand-fold more efficient [46]. The recent structure of the complex between C3d and CR2 [11] shows that SCRs 1 and 2 of CR2 interact with C3d. Preventing or reducing activation of adaptive immunity would be very beneficial for bacteria, especially for ones which cause chronic infections, like *Borrelia*. By superimposing our tripartite structure with the published C3d-CR2 structure (PDB code 3OED) we sought to see, whether FH bound to C3dg prevents binding of CR2. The binding sites for the two proteins are close, but there was no evidence for steric hindrance (S3 Fig).

FHRs have homologous domains with FH19-20 [12] and are known to interact with some microbial proteins [47]. ErpA and ErpP from *B*. *burgdorferi* sensu stricto (strains B31 and LW2, respectively), of which ErpA is highly homologous to OspE of *B*. *burgdorferi* sensu stricto (strain N40), bind FHR2, FHR5 [48] and FHR1 [49]. ErpC, also from *B*. *burgdorferi* sensu stricto, has been shown to bind FHRs 1, 2 and 5 [49, 50]. All three Erps bind full length FH *in vitro*, but when expressed on serum-sensitive *B*. *garinii*, little or no FH binding is seen [48, 50]

The reasons why exogeneously expressed Erp-proteins do not bind FH are unknown. In our tripartite structure, OspE binds simultaneously to C3d and FH20. It might be that *in vivo* Erps from *B*. *burgdorferi* sensu stricto expressed on the surface of *B*. *garinii* are not able to form a similar tripartite structure, which could lead to inadequate protection. Alternatively other, as yet unknown structures on the microbial surface are variable between the species and so affect binding of FH. Even though *in vitro* Erp-proteins seem to have higher affinity for FHRs than for FH [48, 49], the effect of local variations in concentrations of C proteins and their affinities *in vivo* are unknown. Future biochemical and structural studies will show how FHRs *in vivo* affect C activation on the surface of *Borrelia*.

It is tempting to speculate that FHRs have evolved to provide protection against microbes by allowing more efficient C activation [15], which might have led to appearance of new class(es) of FH-binding proteins on microbes. This coevolutionary development could be especially prominent in structurally C sensitive bacteria, like borrelia. It has been shown that, during *Neisseria meningitidis* infection, relative levels of FHR3 (an antagonist for SCRs FH6-7) and FH, together with molecular variations in FH-binding bacterial proteins, affect the outcome of C activation [51]. Overall, functional studies of the possible role of FHRs in microbial evasion of C, especially *in vivo*, are still only preliminary.

**Fig 5:**
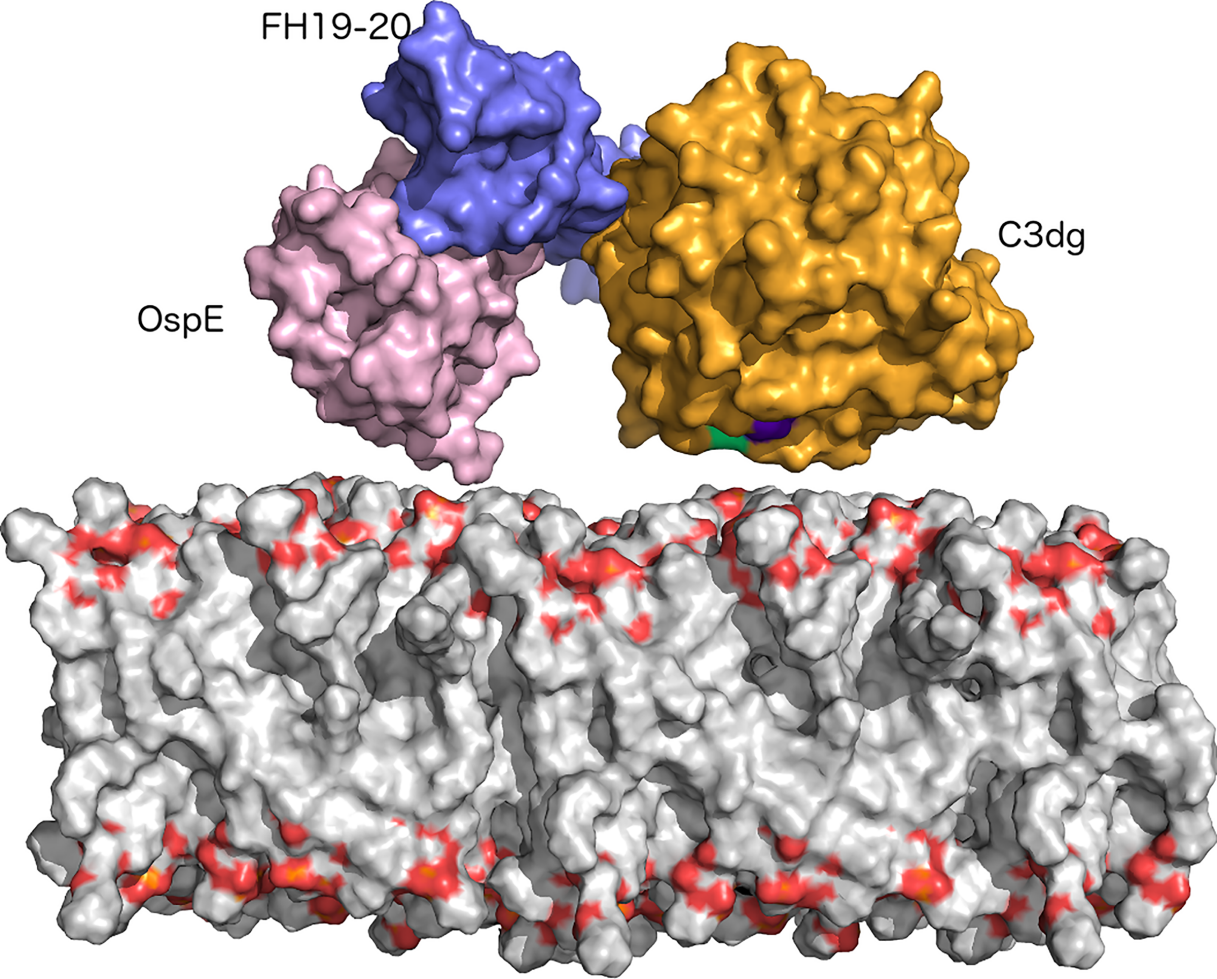
Model of orientation of the trimer on the surface of bacteria. N-terminus of OspE (pink surface model) is facing the surface, as well as the TED-domain of C3dg (orange surface presentation with TED-domain marked in green/purple). FH19-20 (blue surface presentation) can simultaneously bind to both ligands by reposing between the two other proteins.

We report here the crystal structure of the heterotrimer C3dg:FH19-20:OspE at 3.18 Å resolution. This structure describes for the first time on a molecular level how microbial protein, FH19-20 and C3dg interact together to form a tripartite complex. Specific interactions are likely to lead down regulation of the main C opsonin, C3b by FH and evasion the attack and the opsonisation by the AP of complement by an important and common zoonotic pathogen, *Borrelia burgdorferi* sensu stricto, (Fig 5).

### Deposition of coordinates

Coordinates and structure factors have been deposited with Protein Data Bank in Europe (PDBe; https://www.ebi.ac.uk/pdbe/); accession code 5NBQ.

## Supporting information

S1 Fig A σA weighed electron densities of all three trimers. In the panel A trimer ADG, in the panel B trimer BEF and in the panel C trimer CFI. C3dg is orange, FH19-20 blue and OspE purple.
(PDF)

S2 Fig **B-factors of C3d (chains A, B and C), FH19-20 (chains D, E and F) and OspE (chains G and I).** B-factors are plotted to Y-axis (Å^2^) with amino acids on the X-axis. Individual chains are shown using different colors, which are marked to the data labels.
(PDF)

S3 Fig Model of FH19-20 and CR2 bound to C3d. C3d from the trimer CFI (deep orange) and from PDB deposition 3OED (lime green) were aligned using Pymol align–command. Possible contact site between CR2 (salmon red) and FH19 (dark blue) lies on the top, Distance between the residues is app. 3.8Å (measured in Pymol).
(PDF)

S1 Table Primers used in the study. (PDF)

S2 Table Hydrogen bonds between FH19-20 and C3dg in the tripartite structure and compared to previous structure between FH19-20 and C3d (PDB 2XQW). N.d. not detected.
(PDF)

S3 Table Hydrogen bonds between FH19-20 and OspE in the tripartite structure and compared to previous structure between FH19-20 and OspE (PDB 4J38). N.d. not detected.
(PDF)

## Abbreviations

AP: alternative pathway
ASU: asymmetric unit
C: complement
FH: factor H
FHR: Factor H-related protein
HR2: Helsinki Random Screen 2
OspE: outer surface protein E
SCR: short consensus repeat
TED: thioester-containing domain
XDS: X-ray Detector Software
