# Supplementary figures and images for: Crystal structure of a tripartite complex between C3dg, C-terminal domains of factor H and OspE of Borrelia burgdorferi
Source: PLoS One. 2017 Nov 30;12(11):e0188127. doi: 10.1371/journal.pone.0188127 (PMC5708776; doi:10.1371/journal.pone.0188127)

A.

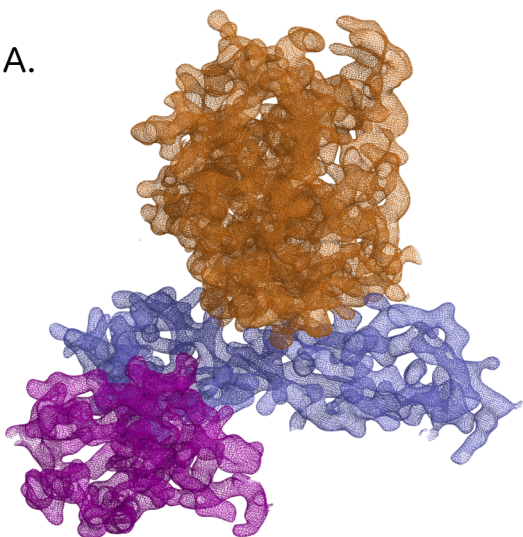

B.

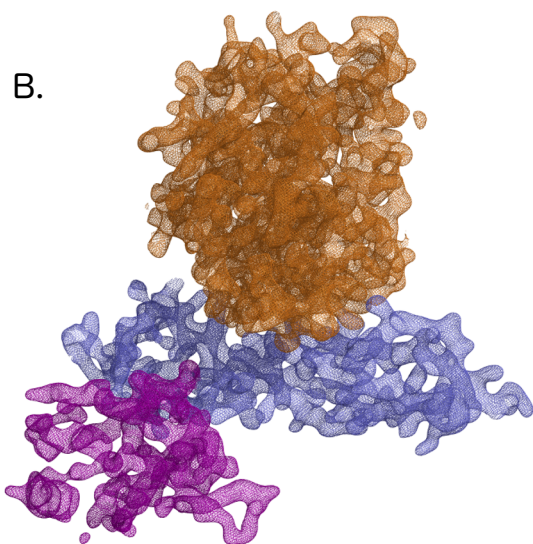

C.

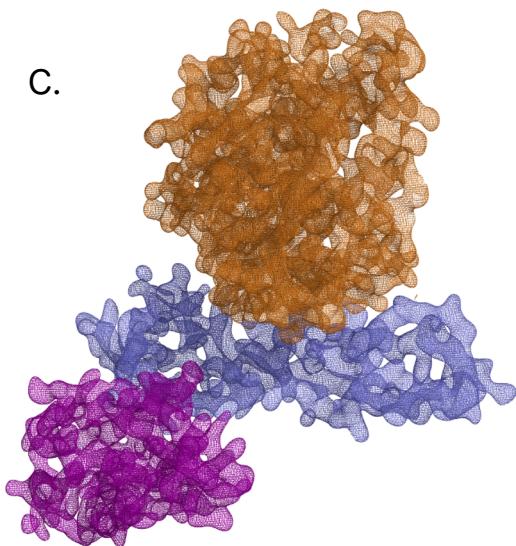

Supplement: S1 Fig — In the panel A trimer ADG, in the panel B trimer BEF and in the panel C trimer CFI. C3dg is orange, FH19-20 blue and OspE purple. (PDF) [file pone.0188127.s001.pdf]

S1 Figure.

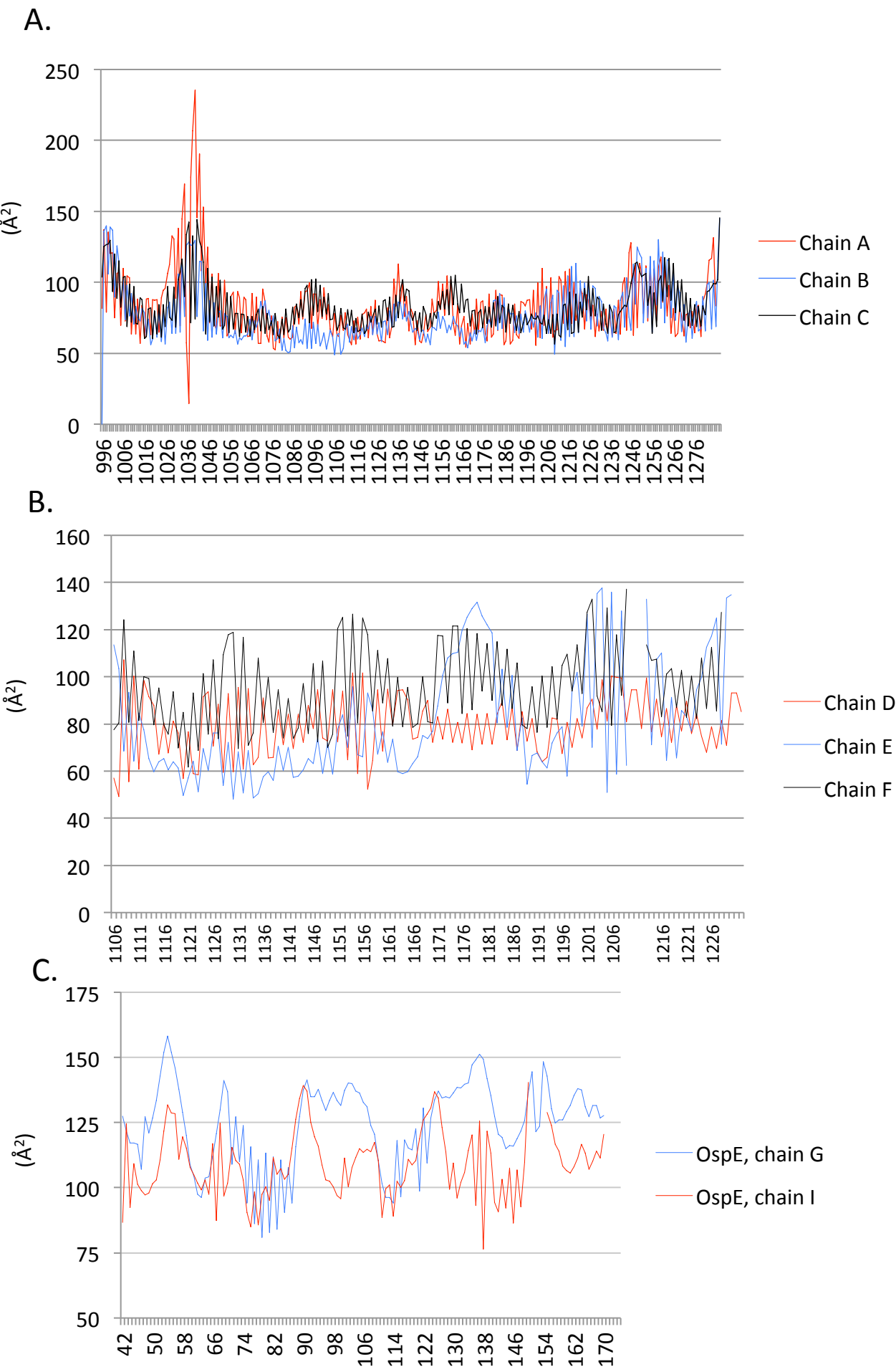

Supplement: S2 Fig — B-factors of C3d (chains A, B and C), FH19-20 (chains D, E and F) and OspE (chains G and I). B-factors are plotted to Y-axis (Å2) with amino acids on the X-axis. Individual chains are shown using different colors, which are marked to the data labels. (PDF) [file pone.0188127.s002.pdf]

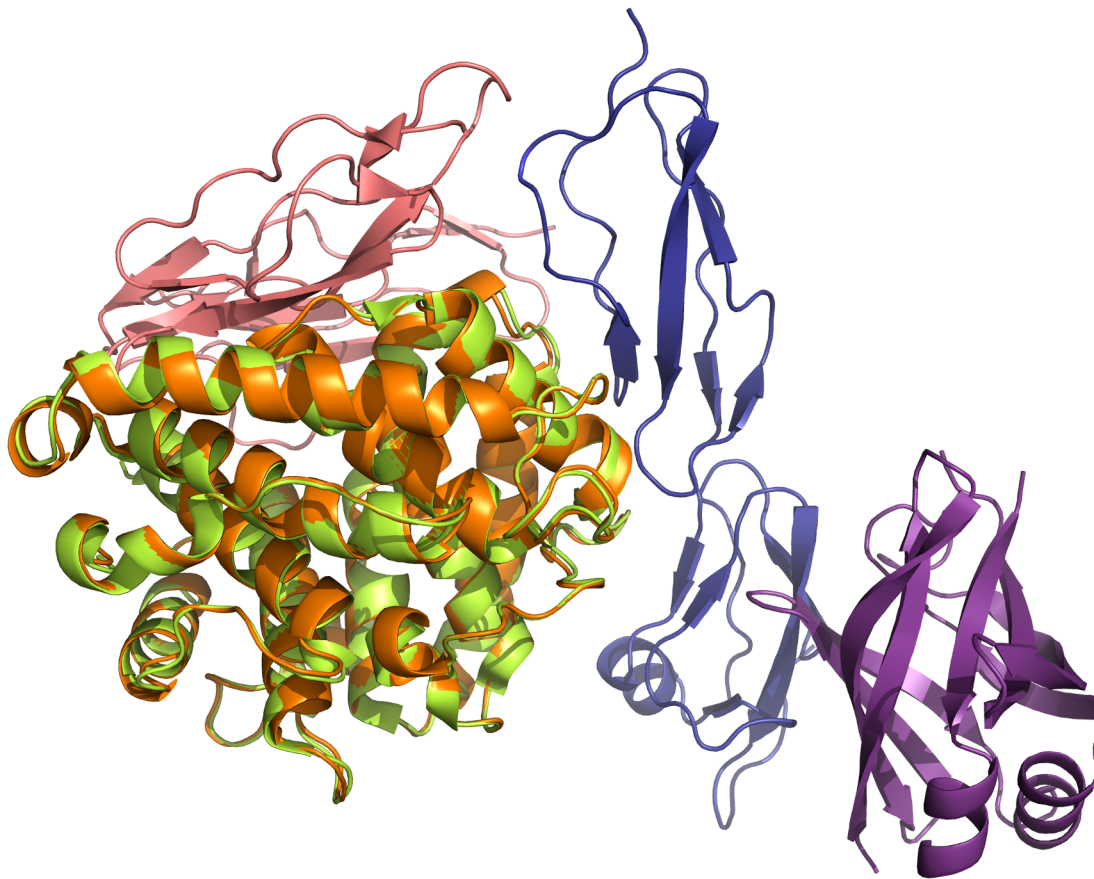

Supplement: S3 Fig — C3d from the trimer CFI (deep orange) and from PDB deposition 3OED (lime green) were aligned using Pymol align–command. Possible contact site between CR2 (salmon red) and FH19 (dark blue) lies on the top, Distance between the residues is app. 3.8Å (measured in Pymol). (PDF) [file pone.0188127.s003.pdf]
