# Supplementary material for: Crystal structure of a tripartite complex between C3dg, C-terminal domains of factor H and OspE of Borrelia burgdorferi
Source: PLoS One. 2017 Nov 30;12(11):e0188127. doi: 10.1371/journal.pone.0188127 (PMC5708776; doi:10.1371/journal.pone.0188127)
Supplement: S1 Table — (PDF) [file pone.0188127.s004.pdf]

S1 Table. Primers used in the study

Primer name	Sequence
C3d-F	CTTTAAGAAGGAGATATACATATGCATCATCATCATCATCACA GCAGCGGCGAAAACCTGTATTTTCAGAGCGACGCGGAACGGCT GAAGCACCTC
C3dg-F	CTTTAAGAAGGAGATATACATATGCATCATCATCATCATCACAG CAGCGGCGAAAACCTGTATTTTCAGAGCGAAGGAGTGCAGAAA GAGGACATCC
C3d/g-R	TCGGGCTTTGTTAGCAGCCGGATCTCAGCGGCTGGGCAGTTGG AGGGACAC
QC_A1153E-F	GTTCTCATCTCGCTGCAGGAAGCTAAAGATATTTGCGAG
QC_A1153E-R	CTCGCAAATATCTTTAGCTTCCTGCAGCGAGATGAGAAC
QC_C1010A-F	GACCCCCTCGGGCGCGGGGGAACAGAAC
QC_C1010A-R	GTTCTGTTCCCCCGCGCCCGAGGGGGTC
C3dg-2F	CTTTAAGAAGGAGATATACATATGGAAGGAGTGCAGAAAGAG GACATCC
C3dg-2R	GGATGTCCTCTTTCTGCACTCCTTCCATATGTATATCTCCTTCTT AAAG
C3d-2F	CTTTAAGAAGGAGATATACATATGGACGCGGAACGGCTGAAGC ACCTC
C3d-2R	GAGGTGCTTCAGCCGTTCCGCGTCCATATGTATATCTCCTTCTT AAAG
C3dg-3F	GTGTCCCTCCAACTGCCCAGCCGCGAAAACCTGTATTTTCAGAG CCATCATCATCATCATCACTGAGATCCGGCTGCTAACAAAGCCC GA
C3dg-3R	TCGGGCTTTGTTAGCAGCCGGATCTCAGTGATGATGATGATGAT GGCTCTGAAAATACAGGTTTTCGCGGCTGGGCAGTTGGAGGGA CAC
C3d-3F	GTGTCCCTCCAACTGCCCAGCCGCGAAAACCTGTATTTTCAGAG CCATCATCATCATCATCACTGAGATCCGGCTGCTAACAAAGCCC GA
C3d-3R	TCGGGCTTTGTTAGCAGCCGGATCTCAGTGATGATGATGATGAT GGCTCTGAAAATACAGGTTTTCGCGGCTGGGCAGTTGGAGGGA CAC
