# Supplementary material for: Crystal structure of a tripartite complex between C3dg, C-terminal domains of factor H and OspE of Borrelia burgdorferi
Source: PLoS One. 2017 Nov 30;12(11):e0188127. doi: 10.1371/journal.pone.0188127 (PMC5708776; doi:10.1371/journal.pone.0188127)
Supplement: S2 Table — N.d. not detected. (PDF) [file pone.0188127.s005.pdf]

S2 Table. Hydrogen bonds between C3dg and FH19-20 in the CFI-interaction.

C3dg residue	Distance (Å)	FH19-20, residue	Analyzed from the crystal structure PDB 2XQW*
Lys^{1106} (N^{ζ})	n.d.	Ile^{1120} (O)	2.83
Lys^{1106} (N^{ζ})	n.d.	Ser^{1122}(O)	3.73
Ile^{1108}(O)	3.16	Gln^{1139} ($N^{\varepsilon 2}$)	
Leu^{1109} (O)	3.36	Asn^{1140} (N)	3.01
Leu^{1109} (O)	3.31	Gln^{1137} ($N^{\varepsilon 2}$)	
Glu^{1110} ($O^{\varepsilon 1}$)	2.85	Gln^{1137} ($N^{\varepsilon 2}$)	3.07
Glu^{1110} (O)	n.d.	Asn^{1140} ($N^{\delta 2}$)	3.83
Gln^{1112} ($O^{\varepsilon 1}$)	3.18	Gln^{1139} ($N^{\varepsilon 2}$)	
Lys^{1113}(N^{ζ})	2.83	Asn^{1140} ($O^{\delta 1}$)	
Asp^{1115} ($O^{\delta 2}$)	2.97	Lys^{1188} (N^{ζ})	2.76
Asp^{1115} ($O^{\delta 1}$)	2.61	Tyr^{1190} (O^{η})	2.61
Asn^{1163} (N)	3.42	Asp^{1119} ($O^{\delta 2}$)	
Ser^{1164} (O^{γ})	2.94	Asp^{1119} ($O^{\delta 2}$)	
Ser^{1164} (N)	3.38	Asp^{1119} ($O^{\delta 2}$)	
Lys^{1171} (N^{ζ})	3.28	Asn^{1117} ($O^{\delta 1}$)	3.49
Lys^{1171} (N^{ζ})	3.16	Gln^{1139} ($O^{\varepsilon 1}$)	
Lys^{1171} (N^{ζ})	3.01	Tyr^{1142} (O^{η})	2.85

* Kajander T, Lehtinen MJ, Hyvärinen S, Bhattacharjee A, Leung E, Isenman DE, et al. Dual interaction of factor H with C3d and glycosaminoglycans in host-nonhost discrimination by complement. PNAS. 2011;108(7):2897-902. doi: 10.1073/pnas.1017087108. PubMed PMID: 21285368; PubMed Central PMCID: PMC3041134.
