# Supplementary material for: Crystal structure of a tripartite complex between C3dg, C-terminal domains of factor H and OspE of Borrelia burgdorferi
Source: PLoS One. 2017 Nov 30;12(11):e0188127. doi: 10.1371/journal.pone.0188127 (PMC5708776; doi:10.1371/journal.pone.0188127)
Supplement: S3 Table — N.d. not detected. (PDF) [file pone.0188127.s006.pdf]

**S3 Table**. Hydrogen bonds between FH20 and OspE in the CFI interaction.

| FH residue | Distance (Å) | OspE residue | Analyzed from the crystal structure PDB 4J38* |
|---|---|---|---|
| $Arg^{1182}$ ($N^{\eta2}$) | 3.10 | $Asp^{73}$ ($O^{\delta2}$) | 3.02 |
| $Arg^{1182}$ ($N^{\eta2}$) | 2.81 | $Glu^{68}$ ($O^{\varepsilon2}$) | 2.97 |
| $Arg^{1182}$ ($N^{\varepsilon}$) | n.d. | $Thr^{84}$ ($O^{\gamma1}$) | 3.75 |
| $Arg^{1182}$ (O) | 2.76 | $Arg^{66}$ ($N^{\eta1}$) | 2.67 |
| $Trp^{1183}$ ($N^{\varepsilon1}$) | n.d. | $Thr^{84}$ ($O^{\gamma1}$) | n.d. |
| $Trp^{1183}$ (O) | 2.81 | $Asn^{77}$ ($N^{\delta2}$) | 3.08 |
| $Thr^{1184}$ ($O^{\delta1}$) | n.d. | $Asn^{77}$ ($O^{\delta1}$) | 3.73 |
| $Ser^{1191}$ (OG ) | 3.10 | $Gly^{80}$ (O) | 2.61 |
| $Glu^{1195}$ ($O^{\varepsilon2}$) | 3.77 | $Tyr^{114}$ ($N^{\zeta}$) | n.d. |
| $Glu^{1195}$ ($O^{\varepsilon2}$) | n.d. | $Gly^{80}$ (N) | 3.70 |
| $Ser^{1196}$ (O) | 3.04 | $Ser^{82}$ (N) | n.d. |
| $Ser^{1196}$ (O) | 3.34 | $His^{81}$ ($N^{\delta1}$) | 3.02 |
| $Ser^{1196}$ ($O^{\gamma}$) | 3.83 | $Val^{120}$ (O) | n.d. |
| $Glu^{1198}$ (N) | 2.81 | $Ser^{82}$ (O) | 2.82 |
| $Glu^{1198}$ ($O^{\varepsilon1}$) | n.d. | $Thr^{84}$ ($O^{\gamma1}$) | 2.90 |
| $Glu^{1198}$ ($O^{\varepsilon1}$) | 3.62 | $Thr^{84}$ (N) | 2.68 |
| $Arg^{1215}$ ($N^{\eta1}$) | 2.88 | $Thr^{84}$ (O) | 3.71 |
| $Arg^{1215}$ ($N^{\varepsilon}$) | 2.92 | $Val^{120}$ (O) | 2.83 |
| $Arg^{1215}$ ($N^{\eta1}$) | n.d. | $Ile^{121}$ (O) | 3.46 |


- Bhattacharjee A, Oeemig JS, Kolodziejczyk R, Meri T, Kajander T, Lehtinen MJ, et al. Structural basis for complement evasion by Lyme disease pathogen Borrelia burgdorferi. J Biol Chem. 2013;288(26):18685-95. doi: 10.1074/jbc.M113.459040. PubMed PMID: 23658013; PubMed Central PMCID: PMC3696643.
